# Recent advances in primary Sjogren's syndrome

**DOI:** 10.12688/f1000research.8352.1

**Published:** 2016-06-17

**Authors:** Nicholas Holdgate, E. Wiliam St.Clair

**Affiliations:** 1 Department of Medicine, Division of Rheumatology and Immunology, School of Medicine, Duke University, Durham, NC, 27710, USA

**Keywords:** Sjögren’s syndrome, inflammatory, rheumatologic diseases

## Abstract

Primary Sjögren’s syndrome, a chronic inflammatory process, is among the most commonly occurring rheumatologic diseases. The clinical hallmark of this disease is exocrine gland dysfunction, resulting predominately in dry eyes and dry mouth. However, the disease often extends beyond the exocrine glands to seriously affect other organs systems, such as the lungs, kidneys, and nervous system. Moreover, patients with primary Sjögren’s syndrome develop non-Hodgkin’s B cell lymphoma at a substantially higher rate than the general population. New research has improved our understanding of disease mechanisms, with notable advances in our knowledge about the genetic susceptibility of disease, the molecular details of the chronic inflammatory response in the salivary glands, and the complex role of the type 1 interferon pathway. The pipeline of drugs under development for the treatment of primary Sjögren’s syndrome is enriched with novel biologics and small molecular entities targeting the pathogenic process. Herein, we summarize the latest advances in elucidating the pathogenesis of primary Sjögren’s syndrome and highlight new drugs in clinical development aiming to reverse the glandular dysfunction and favorably impact the systemic features of this disease.

## Introduction

Despite its prevalence among the rheumatologic diseases, primary Sjögren’s syndrome has attracted less attention from researchers compared to rheumatoid arthritis (RA) and systemic lupus erythematous (SLE), partly owing to a previous misconception that the disease was mostly a nuisance without recognizing that it can threaten the function of vital organ systems. The clinical hallmarks of Sjögren’s syndrome have been well known for many decades and include keratoconjunctivitis sicca, or dry eyes, and xerostomia, or dry mouth. Sjögren’s syndrome is termed secondary when the sicca symptoms occur in association with RA, SLE, or other chronic inflammatory disease. Otherwise, it is termed primary Sjögren’s syndrome. The clinical diagnosis of primary Sjögren’s syndrome is based on the presence of symptoms of dry eyes and dry mouth, often with objective evidence of keratoconjunctivitis sicca and/or decreased salivary flow, and a positive test for serum anti-Ro antibodies or rheumatoid factor or, in the absence of these autoantibodies, a labial salivary gland biopsy showing focal lymphocytic infiltrates ^1^. Reversal of glandular dysfunction and management of extraglandular disease has been hampered by the lack of a proven disease-modifying therapy. Herein, we describe some of the exciting new research about the genetic and immunologic mechanisms of primary Sjögren’s syndrome and the recent progress towards finding a disease-modifying therapy.

Primary Sjögren’s syndrome is among the most common of the systemic autoimmune diseases, ranging in prevalence from 0.1 to 0.6%; it is much more common in women than in men ^2^. The disease is termed an “exocrinopathy” because of its predominant effects on the lacrimal and salivary glands, as well as other exocrine glands in the larynx (hoarseness), trachea (cough), skin (pruritus), and vagina (dyspareunia). In addition to the exocrinopathy, up to 75% of patients with primary Sjögren’s syndrome suffer from extraglandular disease manifestations, and up to 25% develop moderate or severe extraglandular disease ^3^. Extraglandular manifestations range from the less serious complications of purpura, urticaria, inflammatory arthritis, and Raynaud’s syndrome to the more severe end of the spectrum exemplified by renal disease, lung disease, and peripheral neuropathy. Notably, patients with primary Sjögren’s syndrome have an increased risk of developing non-Hodgkin B cell lymphoma, with a recent study showing a cumulative risk at 15 years after diagnosis of 9.8%; this risk is higher than that of patients with RA and SLE who also have an increased risk of developing non-Hodgkin lymphoma ^4, 5^. Interestingly, most non-Hodgkin B cell lymphomas that develop in patients with primary Sjögren’s syndrome belong to the marginal zone histological type and localize to the mucosa-associated lymphoid tissue (MALT), where the underlying disease is most active. The presence of parotid gland enlargement, rheumatoid factor, low C4, cryoglobulinemia, lymphopenia, and higher levels of disease activity as measured by the EULAR Sjögren’s syndrome disease activity index (ESSDAI) predicts a higher lymphoma risk ^6^.

## Current management

In primary Sjögren’s syndrome, the current treatments aim to reduce symptoms of the exocrinopathy as well as control the extraglandular features of the disease. Symptomatic therapies include topical therapies, such as artificial tears, artificial saliva, nasal saline spray, vaginal estrogen cream, and moisturizing skin lotions. Secretagogues, such as sugarless candy and chewing gum, as well as the muscarinic receptor agonists cevimeline and pilocarpine may be used to stimulate tear and saliva production. Topical cyclosporine eye drops are efficacious for improving the signs and symptoms of dry eyes.

At this stage, no disease-modifying drugs have been shown in randomized, placebo-controlled trials to be effective for the treatment of primary Sjögren’s syndrome. While hydroxychloroquine has been employed for reducing the symptoms of fatigue and joint pain in primary Sjögren’s syndrome, it failed in a randomized controlled trial to significantly reduce symptoms of dryness, pain, and fatigue over placebo ^7^. Methotrexate has not been well studied for the treatment of primary Sjögren’s syndrome. In a small open study published 20 years ago, oral weekly methotrexate was not effective in increasing tear or saliva production ^8^. More recently, the tumor necrosis factor (TNF)-α inhibitors infliximab and etanercept have been investigated for their efficacy and safety in patients with primary Sjögren’s syndrome and neither was found to be effective for improving disease outcomes ^9, 10^.

The dearth of proven effective therapies for primary Sjögren’s syndrome has forced clinicians to take an empiric approach to the systemic treatment of extraglandular disease, guided by the severity of organ system involvement. Mild sensory neuropathies, common in patients with primary Sjögren’s syndrome, are generally treated symptomatically with, for example, gabapentin or pregabalin. Recurrent lower extremity purpura caused by a leukocytoclastic vasculitis may usually be managed with support stockings and non-steroidal anti-inflammatory drugs. Systemic corticosteroids may be prescribed for the treatment of organ-threatening vasculitis, non-specific interstitial pneumonitis (NSIP), interstitial nephritis, glomerulonephritis, and motor neuropathies. Other immunosuppressive agents, such as azathioprine, mycophenolate mofetil, and cyclophosphamide, are usually reserved for the treatment of serious or life-threatening extraglandular disease, such as NSIP and glomerulonephritis.

## Advances in understanding of disease pathogenesis

Primary Sjögren’s syndrome results from a complex interplay of several factors, including genetic and epigenetic controls of immune homeostasis and gene expression, age and gender, and environmental insults. It follows a typical multistep model of human autoimmune diseases characterized by loss of immunologic tolerance to self-antigens, the permissive production of autoantibodies, and the subsequent emergence of disease ^11, 12^. Studies of labial salivary glands reveal the contribution of a panoply of cell types, including T cells, B cells, macrophages, dendritic cells, and epithelial cells, that combine to orchestrate a persistent chronic inflammatory response.

The basis for the exocrine gland dysfunction is poorly understood and may result from effects related to the pro-inflammatory cytokine/chemokine milieu, autoantibody-mediated blockade of muscarinic acetylcholine receptors (e.g. anti-muscarinic receptor 3 antibodies), neurotransmitter imbalances, or the destruction of glands. The potential reversibility of glandular dysfunction is unclear and must necessarily await the discovery of a disease-modifying drug that can ameliorate the loss of lacrimal and salivary secretory flow.

### Genetics and epigenetics

Newly discovered genetic associations in primary Sjögren’s syndrome implicate disturbances in both the adaptive and the innate immune pathways ^13, 14^. Genome-wide association and candidate gene studies have identified susceptibility loci in *BLK* and *CXCR5*, genes encoding proteins involved in B cell activation, trafficking, and spatial localization, and in *STAT4*, *IL-12A*, and *IRF5*, genes regulating both adaptive and innate immunity. The finding of risk variants in the *IRF5* gene may explain in part the activation of the type 1 interferon (IFN) pathway in primary Sjögren’s syndrome ^15^, as *IRF5* encodes a transcription factor important for the production of type 1 IFN ^16^. A risk variant of *IRF5*, a CGGGG indel, has been associated with increased levels of *IRF5* transcripts, implying a functional role in the regulation of this pathway ^17^. *STAT4* is triggered by type 1 IFN, interleukin (IL)-12, and IL-23 and when activated leads to the induction of T helper type 1 (TH1) cells and up-regulation of IFN-γ.

Genome-wide association and candidate gene studies have also identified risk loci in the *TNFAIP3* and *TNIP* genes, which regulate nuclear factor kappa-light-chain-enhancer of activated B cells (NFκB) signaling. The potential importance of the *TNFAIP3* locus in the pathogenesis of primary Sjögren’s syndrome is underscored by the finding that *TNFAIP3* knockout mice develop an autoimmune phenotype and have high rates of lymphoma ^18^. Moreover, a study of patients with primary Sjögren’s syndrome from a French cohort has recently found germline mutations in the coding region of *TNFAIP3* (A20) that are associated with an increased risk for lymphoma ^19^. Interestingly, in some instances, the samples of lymphoma tissue harbored the same germline mutations in *TNFAIP3* that were associated with an increased risk for lymphoma development, while in other cases without the germline mutation, the lymphoma tissue had somatic mutations in *TNFAIP3*. Some of these *TNFAIP3* variants impaired control of NFκB activation, suggesting a possible escape mechanism for transition into lymphoma ^19^.

The epigenetic regulation of gene expression in primary Sjögren’s syndrome has not been studied until recently. Patients with primary Sjögren’s syndrome have an increase in hypomethylation of the *STAT4*, *IL-12A*, *IRF5*, *BLK*, *CXCR5*, and *TNIP* genes ^20, 21^, suggesting a possible role for epigenetic regulation in the pathogenesis of this disease.

### Adaptive immunity

The role of B cells in the pathogenesis of primary Sjögren’s syndrome is strongly implied by the association of this disease with autoantibody production, B cell hyperactivity, germinal center formation in the target tissue, and lymphomagenesis. In primary Sjögren’s syndrome, it is not known if pathologic B cell activation occurs in the spleen and lymph nodes or the germinal center-like structures (tertiary lymphoid tissue) of the target tissues, or both. T-cell-dependent antigens that activate B cells rely on T follicular helper (TFH) cells, which stimulate the formation and maintenance of germinal centers through the expression of CD154 (CD40 ligand) on the cell surface and the secretion of IL-4 and IL-21; they mediate the selection and survival of B cells that differentiate into antibody-secreting plasma cells and memory B cells. TFH cells have been identified in labial salivary gland tissue, predominantly within organized structures ^22^. The differentiation of CD4+ naïve T cells into TFH cells may be promoted by the glandular epithelium. Salivary gland epithelial cells expressing IL-6 and inducible T cell co-stimulator ligand (ICOS-L) have been shown in culture to stimulate the differentiation of CD4+ naïve T cells into IL-21-secreting TFH cells ^23^.

Controlling TFH cell-mediated B cell activation may be a useful therapeutic strategy in primary Sjögren’s syndrome. Two experimental therapies, abatacept and rituximab, have been shown to reduce the absolute number of circulating TFH cells ^24^, but the significance of these findings is unclear, since neither of these agents has been proven effective in randomized, controlled trials involving patients with primary Sjögren’s syndrome. In the future, inhibiting the differentiation of TFH cells by neutralizing IL-21 or IL-6 or blocking the interaction between ICOS and ICOS-L, either alone or in combination, may also be therapeutic strategies worth pursuing.

B-cell-activating factor (BAFF), a member of the TNF receptor family, is a potent activator of B cells. BAFF is the natural ligand of three receptors, BAFF-R (BR3), a transmembrane activator and calcium modulator, as well as transmembrane activator and calcium-modulator and cyclophilin ligand interactor (TACI) and B cell maturation antigen (BCMA). Several lines of evidence implicate BAFF in the pathogenesis of primary Sjögren’s syndrome. First, compared with healthy controls, patients with primary Sjögren’s syndrome have higher serum levels of BAFF and show increased expression of BAFF in labial salivary glands ^25^. Second, BAFF transgenic mice exhibit a Sjögren’s syndrome phenotype characterized by infiltration of lacrimal and salivary glands with lymphocytes that include a unique population of marginal zone B cells ^26^. Third, salivary gland epithelial cells from patients with primary Sjögren’s syndrome also express BAFF; in addition, type 1 IFN, which is up-regulated in both blood and salivary gland tissue from patients with primary Sjögren’s syndrome, is a potent stimulator of BAFF expression ^25, 27^. BAFF engagement with its receptor, BR3, has been shown to activate the phosphatidylinositol 3-kinase delta isoform (PI3kδ), a signaling molecule involved in the regulation of cell growth, proliferation, differentiation, motility, survival, and intracellular trafficking of B cells, as well as lymphomagenesis ^28^. For these reasons, the BAFF/BR3 pathway is a promising therapeutic target in primary Sjögren’s syndrome.

The lymphotoxin β receptor (LTβR) is part of the signaling system that directs stromal cells to differentiate into specialized vasculature and regulates certain reticular networks that guide and position cells for optimal encounters with antigen ^29^. Blockade of the LTβR system in mice reduces addressin expression on high endothelial venules (HEVs) and thereby inhibits entry of lymphocytes into lymph nodes and mucosal environments, resulting in a circulating lymphocytosis ^30^. In male NOD mice, LTβR-Ig treatment reduces glandular inflammation, blocks HEV formation, and partially restores salivary flow ^31^. In a phase II trial, baminercept (LTβR-Ig) treatment was ineffective in decreasing the signs and symptoms of RA; however, it was shown to increase the blood lymphocyte counts and reduce the whole blood IFN signature ^32^. Although inhibiting the lymphotoxin pathway has a sound theoretical basis for treating primary Sjögren’s syndrome, preliminary results from a randomized, placebo-controlled 24-week trial showed no significant differences between the baminercept and placebo groups in the change in stimulated salivary flow or ESSDAI ^33^.

### Innate immunity

Viruses have long been considered etiologic factors in primary Sjögren’s syndrome. They have been receiving more attention of late as a possible disease trigger because of the association between primary Sjögren’s syndrome and up-regulation of the type 1 IFN pathway, an innate immune mechanism of antiviral host defense. An IFN signature has been detected in the peripheral blood of 65% of patients with primary Sjögren’s syndrome ^34^ and can be observed in labial salivary gland tissue from patients with this disease ^34^. The majority of the IFN inducible genes are up-regulated by both IFN-α and IFN-γ, while some are uniquely due to the effects of IFN-α or IFN-γ. In the labial salivary glands, there is heterogeneity among patients in the expression of IFN-α and IFN-γ inducible genes, with higher focus scores associated with predominant IFN-γ effects ^34, 35^.

To protect against viral infection, the host innate immune system has evolved sensors of nucleic acids. The same nucleic acid sensors that defend against viruses may also contribute to the pathogenesis of autoimmune diseases. The two main systems for detecting nucleic acids are the Toll-like receptors (TLRs) and the cytosolic RNA and DNA sensors. The endosomal TLRs mainly detect nucleic acids, such as double-stranded RNA (TLR3), single-stranded RNA (TLR7), and double-stranded DNA (TLR9) ^36^. Through an autoantibody-dependent, FcγR-mediated process, nucleic acids gain access to the cytosol and in turn ligate endosomal TLRs, which activate dendritic cells and macrophages to produce type 1 IFN ^37^. Two other key intracellular sensors of RNA and DNA may also play a role in the pathogenesis of autoimmune diseases such as primary Sjögren’s syndrome, namely the retinoic acid-inducible gene I (RIG-I)-like receptors RIG-I and Mda5 and cyclic GMP-AMP synthase/STING, respectively, which also activate the production of type 1 IFN ^38^. It has been theorized that the evolutionary pressures to produce a more robust antiviral response may also tip the balance towards developing an autoimmune disease.

Epstein-Barr virus (EBV) is a promising candidate for triggering autoimmune disease owing to its ability to infect B cells and promote the survival of autoreactive B cell clones ^39^. A human DNA γ-herpesvirus, it establishes an asymptomatic latent infection in B cells and epithelial cells. EBV-encoded small RNA (EBER), the most abundant transcripts in latently infected cells, contribute to EBV-mediated pathogenesis by activating RIG-I and stimulating innate immune signaling through TLR3 ^40^. EBERs also bind to the La protein and are secreted outside the cell in exosomes, where they may bind to TLR3 and activate dendritic cells ^41^. However, studies comparing the expression of EBV nucleic acids and proteins in the salivary glands between patients with primary Sjögren’s syndrome and healthy controls have produced conflicting results.

Croia and colleagues recently attempted to settle this controversy by focusing their search for latent EBV infection on labial salivary gland tissue with germinal center-like structures expressing the mRNA for activation-induced cytidine deaminase (AID) and CD21 ^42^. AID is involved in somatic hypermutation, gene conversion, and class-switch recombination of immunoglobulin genes, while CD21 is a marker of follicular dendritic cells. They found that EBERs were preferentially expressed in the AID ^+^CD21 ^+^ labial salivary gland biopsies compared to AID ^-^CD21 ^-^ samples from the disease patients and healthy controls, with EBERs localized mostly to the follicular B cells and plasma cells. Further evidence of EBV latency was obtained by detection of the latent EBV antigen LMP-2 in the B cells within the ectopic follicles. Notably, the lytic-phase antigen BFRF1 was expressed in the perifollicular plasma cells with Ro52 autoreactivity, a prominent autoantigen in primary Sjögren’s syndrome. Whether EBER transcripts from latent EBV infection are contributing to the inflammatory response in primary Sjögren’s syndrome is an open question, but the results of this study draw more attention to this possibility.

Retrotransposons comprise approximately 40% of the human genome and include the retrovirus long-terminal repeats (LTRs), members of the ‘long interspersed nuclear element 1’ (LINE-1, or L1) family, and ‘short interspersed nuclear elements’ (SINEs) ^39^. Most human SINEs are ‘Alu’ elements, which are capable of replicating themselves by hijacking the L1 reverse transcriptase ^39^. A recent study found that anti-Ro60–positive SLE immune complexes containing Alu RNAs and Alu transcripts were up-regulated in whole blood samples from patients with SLE relative to control samples and established that aberrant expression of endogenous Alu RNAs stimulated a TLR7-dependent response that enhances the secretion of IFN-α, IL-6, and TNF-α ^41^. The results from these studies suggest that retroelements may stimulate autoimmunity by amplifying immune responses through endogenous RNA sensors ^41^. There is also evidence that endogenous retroviruses and innate sensing pathways are integral to T cell-independent B cell activation ^43^. Thus, if retroelements play a significant role in the pathogenesis of primary Sjögren’s syndrome, then decreasing the abundance of nucleic acids inside cells or increasing the threshold of their intracellular sensors may represent a new strategy for treating this disease.

Natural killer (NK) cells are innate lymphoid cells that play an important role in antiviral host defense and are increasingly implicated in the pathogenesis of autoimmune disease ^44^. Human NK cells are CD3 ^-^ and separated into two major groups by their expression of CD16 and CD56. The CD56 ^dim^ NK cells that comprise approximately 90% of the circulating NK cells express high levels of CD16, inhibitory killer-like receptors (KIRs), and perforin; CD56 ^bright^ NK cells express low levels of CD16, KIRs, and perforin and are abundant in secondary lymphoid tissues where they modulate immune responses through their secretion of cytokines and chemokines ^44^.

NKp30, a NK cell-specific activating receptor, regulates cross-talk between NK and dendritic cells and mediates secretion of IL-12 and IFN-γ. In a large cohort of patients with primary Sjögren’s syndrome, genetic polymorphisms within the promoter region of the *NKp30* gene were found to be associated with decreased disease susceptibility and reduced levels of gene expression and function of the NKp30 protein ^45^. In this study, circulating NK cells from the patients with primary Sjögren’s syndrome expressed higher levels of *NKp30* than controls and showed increased secretion of IFN-γ ^45^. Moreover, excessive numbers of NK cells were found to be present in the labial salivary gland tissue from patients with primary Sjögren’s syndrome, localizing outside inflammatory foci, and nearby glandular epithelial cells expressing B7H6, the ligand for NKp30 ^45^. It is possible that NKp30 ^+^ cells may be an important source of IFN-γ in the target organ of primary Sjögren’s syndrome through the engagement of B7H6 on epithelial cells.

IL-22, a member of the IL-1 family, is produced by a variety of cell types, including TH17 cells, γδ T cells, NK T cells, and innate lymphoid cells (ILCs); it mainly acts on nonhematopoietic cells such as epithelial cells ^46^. Increased expression of IL-22 has been linked to the pathogenesis of tissue inflammation and regeneration of epithelial tissues following injury ^46^. Therefore, IL-22 exerts both pro-inflammatory and protective effects and its function has been shown to be tissue and context dependent. IL-22, IL-23, and IL-17 mRNA and protein levels are up-regulated in labial salivary gland tissue from patients with primary Sjögren’s syndrome compared to healthy controls ^47^.

In labial salivary glands from patients with primary Sjögren’s syndrome, IL-22 has been shown to be produced by activated dendritic cells, T cells, and the NKp44+ subset of NK cells ^47^. Further insights into the possible role of IL-22 have been investigated in a virus-induced mouse model of Sjögren’s syndrome, where IL-22 was found to be mainly produced in the early stages of infection by γδ T cells and then later by αβ T cells, with lesser amounts coming from ILCs and NK cells ^48^. In this study, IL-22/IL-22R engagement was needed for enhanced expression of CXCL12 and CXCL13, recruitment of B cells, and organization of tertiary lymphoid tissue. Although IL-22 may be a potential therapeutic target in primary Sjögren’s syndrome, its role in promoting tissue regeneration and restoring barrier function after tissue injury may preclude systemic IL-22 blockade over long periods of time.

## Investigational systemic therapies

The pipeline of investigational therapies for primary Sjögren’s syndrome has grown substantially over the past 5 years ( Table 1). Despite the initial promise of rituximab therapy for this disease, the results of two randomized, placebo-controlled trials from France and the United Kingdom recently failed to demonstrate significant improvement in the endpoints of dryness, pain, and fatigue ^49, 50^. Other targeted approaches are in much earlier phases of development and have not been adequately studied in randomized, controlled trials. Open treatment with belimumab, a monoclonal antibody that inhibits BAFF, led to some improvement in physician assessment of systemic disease activity and serologic markers of B cell function but failed to show any significant benefit in terms of reducing symptoms of fatigue, pain, and dryness ^51^.

**Table 1.** Biologic therapies under development in primary Sjögren’s syndrome*

Drug	Target	Mechanism of Action	Phase of Study
UCB5857	PI3Kδ	Selective inhibitor of PI3Kδ preventing transmission of cell surface receptor signaling	II
CFZ533	CD40	Fc silent antibody to CD40 preventing B cell stimulation and differentiation without depletion	II
AMG557	ICOS	Inhibit activation of TFH	II
VAY736	BAFF-R	Antibody to BAFF-R preventing BAFF-mediated B cell proliferation and survival	II
Low-dose IL-2	CD4 ^+^CD25 ^+^ T cells	Low-dose interleukin 2 expands Treg cells	II
Rituximab + belimumab	CD20 B cells, BAFF	Anti-CD20-dependent depletion of B cells combined with BAFF blockade to decrease survival of self-reactive B cells	II
Tocilizumab	IL-6R	Blockade of IL-6R preventing IL-6-dependent TH17 and TFH cell differentiation	II
Abatacept	CD80/86	CTLA4-Ig binding of CD80/86 prevents co-stimulation-dependent activation of CD4 T Cells	III

* From clinicaltrials.gov Abbreviations: BAFF, B-cell-activating factor; BAFF-R, B-cell-activating factor receptor; CTLA4-Ig, cytotoxic T-lymphocyte-associated protein 4 – Ig fusion protein (abatacept); ICOS, inducible T cell costimulator; IL-6, interleukin-6; IL-6R, interleukin-6 receptor; PI3Kδ, phosphatidylinositol-4,5-bisphosphate 3-kinase delta; TFH, T follicular helper cell; TH17, T helper 17 cell; Treg cell, T regulatory cell

A clinical trial has been initiated in patients with primary Sjögren’s syndrome that combines treatment with both rituximab and belimumab. It is based on the rationale that BAFF levels increase following rituximab-induced CD20 B cell depletion ^52^, which in turn may promote the survival of self-reactive B cells and perpetuate the breach in B cell tolerance. In theory, combining belimumab with a B-cell-depleting agent would neutralize the excessive BAFF and allow for reconstitution of a non-self-reactive B cell repertoire. Other inhibitors of the BAFF/BR3 pathway are under development as well. Since PI3kδ is a signaling molecule crucial for BR3 function ^28^, studies are also now underway in patients with primary Sjögren’s syndrome to investigate the potential benefits of a small molecule PI3kδ inhibitor.

Bruton’s tyrosine kinase (Btk) inhibitors, which are approved for the treatment of certain B cell malignancies, are also in early stage clinical trials for the treatment of primary Sjögren’s syndrome. These small molecule Btk inhibitors can not only inhibit B cell receptor-dependent B cell proliferation and activation but also impact FcR-dependent proinflammatory cytokine production by myeloid cells ^53^.

The use of co-stimulatory blockade with abatacept is a well-reasoned therapeutic intervention for primary Sjögren’s syndrome owing to the important role of T cell activation in the pathogenesis of this disease. In an open-label study, treatment with abatacept was shown to significantly decrease the scores for the ESSDAI and EULAR Sjögren’s syndrome patient-reported index (ESSPRI) and the serum levels of rheumatoid factor and IgG ^54^. However, measures of salivary and lacrimal gland function did not change during treatment ^52^. Larger controlled studies of abatacept therapy for primary Sjögren’s syndrome are in progress.

Several biologics are under development for the treatment of primary Sjögren’s syndrome that inhibit the activation of T cells. A non-depleting, Fc-silent, anti-CD40 monoclonal antibody (CFZ533) blocks the binding of CD40 to CD154 and thereby inhibits T cell activation. CD154 is particularly important in the activation of TFH cells. TFH cells are also being targeted by a monoclonal antibody to B7RP1 (ICOS-L), or AMG557. Blocking the B7RP1/ICOS-L pathway has been shown to decrease the differentiation of CD4 ^+^ naïve T cells into TFH cells and inhibit germinal center formation ^55^ and thus could decrease B cell activation and ectopic lymphoid tissue formation in the target organs of primary Sjögren’s syndrome.

Tocilizumab, an IL-6 receptor-blocking antibody, has been approved for the treatment of RA and juvenile idiopathic arthritis and is currently in a phase II study investigating its efficacy and safety in primary Sjögren’s syndrome. The IL-6/IL-6 receptor pathway is important in promoting the differentiation and maintenance of TH17 and TFH cells. Low-dose IL-2 therapy is also being considered as a possible therapy for primary Sjögren’s syndrome because of its capacity to expand the T regulatory cell population ^56, 57^; however, IL-2 can also have the undesirable effect of activating T cells and perpetuating the effector arm of a T-cell-driven response. Whether the dose of IL-2 can be adjusted to favor the expansion of T regulatory cells over the activation of pathogenic effector T cells will require further study.

IFN-α is an intriguing therapeutic target in primary Sjögren’s syndrome for the reasons described above. Sifalimumab and rontalizumab, anti-IFN-α monoclonal antibodies under development for the treatment for SLE, would also have a strong rationale for testing in primary Sjögren’s syndrome. In addition, anti-retroviral therapies might have some therapeutic appeal in the future considering the emerging evidence that expression of endogenous retroelements in the target tissue may engage nucleic acid sensors and augment chronic inflammatory responses.

## Looking forward

Drug developers are gradually shifting their attention towards primary Sjögren’s syndrome as a possible indication for new targeted therapies. The prevalence of primary Sjögren’s syndrome and the ready access to the target tissue through labial salivary gland biopsy provides unique advantages for investigation. Moreover, the lack of an approved disease-modifying therapy affords appropriate equipoise for conducting placebo-controlled trials of experimental therapies without the concomitant use of background immunosuppressive agents.

There are also challenges ahead in bringing a new drug through the development process owing to the lack of a roadmap for success. Many failed drug trials in primary Sjögren’s syndrome have raised questions about the optimal approach for investigating new therapeutics in the future. Thus far, the focus has been on interventions that are immunomodulatory and that down-regulate chronic inflammatory responses. Other mechanisms may be at play, such as neuroendocrine abnormalities, that affect some of the disease manifestations, such as fatigue. There is uncertainty about the most appropriate selection of subjects for clinical trials. Some authorities have advocated for the inclusion of patients with early disease only (e.g. < 4 years from diagnosis) with a minimum cut-off for stimulated whole salivary flow to ensure participants have sufficient glandular secretory capacity to detect a treatment response. This approach is logical if the experimental treatment is aimed at improving glandular function. Other trials may be designed to focus on the subset of patients with moderate-to-high levels of systemic disease activity if the investigational drug is hypothesized to benefit patients with extraglandular manifestations of disease.

What is the appropriate primary endpoint for evaluating the clinical efficacy of a targeted therapy? Is it unstimulated or stimulated salivary flow? The answer is probably “yes” if increasing tear and salivary flow is the postulated action of the experimental agent. If the therapy is aimed at other disease manifestations, then two composite indices, the ESSPRI and ESSDAI, have been developed that broadly quantify disease activity. ESSPRI is an instrument that measures patient-oriented outcomes such as dryness, fatigue and pain symptoms, while the ESSDAI is a validated index of systemic disease activity. Recent work has set thresholds for clinically meaningful improvement in the ESSPRI (decrease in one point or 15%) and ESSDAI (≥ three points) ^58^. It has been proposed that an ESSDAI of five points or more (moderate disease activity) should be an eligibility requirement for evaluating new therapies directed at systemic manifestations of primary Sjögren’s syndrome ^58^. However, applying these restricted inclusion criteria significantly reduces the proportion of patients with primary Sjögren’s syndrome that would be eligible for a clinical trial ^59, 60^. Regardless, a more focused approach of testing new drugs in selected study populations with outcomes based on the mechanistic rationale of the experimental drug may improve the success rate of clinical trials in this disease.

The accelerated pace of discovery in primary Sjögren’s syndrome promises to illuminate the genetic, epigenetic, and environmental factors that are responsible for triggering and perpetuating the disease process. This new knowledge will inform the development of novel therapies that in the future will positively impact the care of patients who suffer from this chronic illness.

## Abbreviations

AID, activation-induced cytidine deaminase; BAFF, B-cell-activating factor; BAFF-R, B-cell-activating factor receptor; BCMA, B cell maturation antigen; Btk, Bruton’s tyrosine kinase; EBV, Epstein-Barr virus; EBER, EBV encoded small RNA; ESSDAI, EULAR Sjögren’s syndrome disease activity index; ESSPRI, EULAR Sjögren’s syndrome patient-reported index; HEVs, high endothelial venules; ICOS, inducible T cell co-stimulator; ICOS-L, ICOS ligand; IFN, interferon; IL, interleukin; ILC, innate lymphoid cell; KIR, killer-like receptor; LINE-1/L1, long interspersed nuclear element 1; LTβR, lymphotoxin β receptor; LTRs, long-terminal repeats; MALT, mucosa-associated lymphoid tissue; NFκB, nuclear factor kappa-light-chain-enhancer of activated B cells; NK, natural killer; NSIP, non-specific interstitial pneumonitis; PI3kδ, phosphatidylinositol 3-kinase delta; RA, rheumatoid arthritis; RIG-1, retinoic acid-inducible gene 1; SINEs, short interspersed nuclear elements; SLE, systemic lupus erythematous; TACI, transmembrane activator and calcium-modulator and cyclophilin ligand interactor; TFH, T follicular helper; TH, T helper; TNF, tumor necrosis factor; TLR, Toll-like receptor.

## References

[1] Rasmussen A Ice JA Li H: Comparison of the American-European Consensus Group Sjogren's syndrome classification criteria to newly proposed American College of Rheumatology criteria in a large, carefully characterised sicca cohort. *Ann Rheum Dis.* 2014;73(1):31–8. 10.1136/annrheumdis-2013-203845 23968620 PMC3855629

[2] Bowman SJ Ibrahim GH Holmes G: Estimating the prevalence among Caucasian women of primary Sjögren's syndrome in two general practices in Birmingham, UK. *Scand J Rheumatol.* 2004;33(1):39–43. 10.1080/03009740310004676 15124941

[3] Baldini C Pepe P Quartuccio L: Primary Sjogren's syndrome as a multi-organ disease: impact of the serological profile on the clinical presentation of the disease in a large cohort of Italian patients. *Rheumatology (Oxford).* 2014;53(5):839–44. 10.1093/rheumatology/ket427 24369420

[4] Solans-Laqué R López-Hernandez A Bosch-Gil JA: Risk, predictors, and clinical characteristics of lymphoma development in primary Sjögren's syndrome. *Semin Arthritis Rheum.* 2011;41(3):415–23. 10.1016/j.semarthrit.2011.04.006 21665245

[5] Nocturne G Mariette X: Sjögren Syndrome-associated lymphomas: an update on pathogenesis and management. *Br J Haematol.* 2015;168(3):317–27. 10.1111/bjh.13192 25316606

[6] Nocturne G Virone A Ng WF: Rheumatoid Factor and Disease Activity Are Independent Predictors of Lymphoma in Primary Sjögren's Syndrome. *Arthritis Rheumatol.* 2016;68(4):977–85. 10.1002/art.39518 26606524

[7] Gottenberg JE Ravaud P Puéchal X: Effects of hydroxychloroquine on symptomatic improvement in primary Sjögren syndrome: the JOQUER randomized clinical trial. *JAMA.* 2014;312(3):249–58. 10.1001/jama.2014.7682 25027140

[8] Skopouli FN Jagiello P Tsifetaki N: Methotrexate in primary Sjögren's syndrome. *Clin Exp Rheumatol.* 1996;14(5):555–8. 8913659

[9] Mariette X Ravaud P Steinfeld S: Inefficacy of infliximab in primary Sjögren's syndrome: results of the randomized, controlled Trial of Remicade in Primary Sjögren's Syndrome (TRIPSS). *Arthritis Rheum.* 2004;50(4):1270–6. 10.1002/art.20146 15077311

[10] Moutsopoulos NM Katsifis GE Angelov N: Lack of efficacy of etanercept in Sjögren syndrome correlates with failed suppression of tumour necrosis factor alpha and systemic immune activation. *Ann Rheum Dis.* 2008;67(10):1437–43. 10.1136/ard.2007.077891 18198195

[11] Brennan MT Sankar V Leakan RA: Sex steroid hormones in primary Sjögren's syndrome. *J Rheumatol.* 2003;30(6):1267–71. 12784401

[12] Kivity S Arango MT Ehrenfeld M: Infection and autoimmunity in Sjogren's syndrome: a clinical study and comprehensive review. *J Autoimmun.* 2014;51:17–22. 10.1016/j.jaut.2014.02.008 24637076

[13] Lessard CJ Li H Adrianto I: Variants at multiple loci implicated in both innate and adaptive immune responses are associated with Sjögren's syndrome. *Nat Genet.* 2013;45(11):1284–92. 10.1038/ng.2792 24097067 PMC3867192

[14] Li Y Zhang K Chen H: A genome-wide association study in Han Chinese identifies a susceptibility locus for primary Sjögren's syndrome at 7q11.23. *Nat Genet.* 2013;45(11):1361–5. 10.1038/ng.2779 24097066

[15] Miceli-Richard C Comets E Loiseau P: Association of an *IRF5* gene functional polymorphism with Sjögren's syndrome. *Arthritis Rheum.* 2007;56(12):3989–94. 10.1002/art.23142 18050197 PMC3184606

[16] Takaoka A Yanai H Kondo S: Integral role of IRF-5 in the gene induction programme activated by Toll-like receptors. *Nature.* 2005;434(7030):243–9. 10.1038/nature03308 15665823

[17] Miceli-Richard C Gestermann N Ittah M: The CGGGG insertion/deletion polymorphism of the *IRF5* promoter is a strong risk factor for primary Sjögren's syndrome. *Arthritis Rheum.* 2009;60(7):1991–7. 10.1002/art.24662 19565491

[18] Lee EG Boone DL Chai S: Failure to regulate TNF-induced NF-kappaB and cell death responses in A20-deficient mice. *Science.* 2000;289(5488):2350–4. 10.1126/science.289.5488.2350 11009421 PMC3582399

[19] Nocturne G Tarn J Boudaoud S: Germline variation of TNFAIP3 in primary Sjögren's syndrome-associated lymphoma. *Ann Rheum Dis.* 2016;75(4):780–3. 10.1136/annrheumdis-2015-207731 26338037

[20] Imgenberg-Kreuz J Sandling JK Carlsson Almlof J: Hypomethylation in Enhancer and Promoter Regions of Interferon Regulated Genes in Multiple Tissues Is Associated with Primary Sjögren’s Syndrome.[Abstract 2100]. *Arthritis Rheumatol.* 2015;67(suppl 10). Reference Source

[21] Cole M Quach D Quach HL: Genome-Wide DNA Methylation Signatures of Salivary Gland Inflammation in Sjogren’s Syndrome.[Abstract 1257]. *Arthritis Rheumatol.* 2015;67(suppl 10). Reference Source 10.1002/art.39792 PMC5132022 27332624

[22] Szabo K Papp G Dezso B: The histopathology of labial salivary glands in primary Sjögren's syndrome: focusing on follicular helper T cells in the inflammatory infiltrates. *Mediators Inflamm.* 2014;2014: 631787. 10.1155/2014/631787 25177110 PMC4142299

[23] Gong YZ Nititham J Taylor K: Differentiation of follicular helper T cells by salivary gland epithelial cells in primary Sjögren's syndrome. *J Autoimmun.* 2014;51:57–66. 10.1016/j.jaut.2013.11.003 24411167

[24] Verstappen GM Meiners PM Corneth OB: Treatment with Abatacept or Rituximab Targets T Follicular Helper Cells in Patients with Primary Sjogren s Syndrome.[Abstract 3205]. *Arthritis Rheumatol.* 2015;67(suppl 10). Reference Source

[25] Groom J Kalled SL Cutler AH: Association of BAFF/BLyS overexpression and altered B cell differentiation with Sjögren's syndrome. *J Clin Invest.* 2002;109(1):59–68. 10.1172/JCI14121 11781351 PMC150825

[26] Chiorini JA Cihakova D Ouellette CE: Sjögren syndrome: advances in the pathogenesis from animal models. *J Autoimmun.* 2009;33(3–4):190–6. 10.1016/j.jaut.2009.09.009 19800762 PMC3439154

[27] Ittah M Miceli-Richard C Gottenberg JE: Viruses induce high expression of BAFF by salivary gland epithelial cells through TLR- and type-I IFN-dependent and -independent pathways. *Eur J Immunol.* 2008;38(4):1058–64. 10.1002/eji.200738013 18350548

[28] Nayar S Campos J Buckley C: Phosphatidylinositol-3-Kinase Delta Pathway a Novel Therapeutic Target for Sjogren’s Syndrome.[Abstract 1053]. *Arthritis Rheumatol.* 2015;67(suppl 10). Reference Source

[29] Browning JL: Inhibition of the lymphotoxin pathway as a therapy for autoimmune disease. *Immunol Rev.* 2008;223(1):202–20. 10.1111/j.1600-065X.2008.00633.x 18613838

[30] Browning JL Allaire N Ngam-Ek A: Lymphotoxin-beta receptor signaling is required for the homeostatic control of HEV differentiation and function. *Immunity.* 2005;23(5):539–50. 10.1016/j.immuni.2005.10.002 16286021

[31] Gatumu MK Skarstein K Papandile A: Blockade of lymphotoxin-beta receptor signaling reduces aspects of Sjögren's syndrome in salivary glands of non-obese diabetic mice. *Arthritis Res Ther.* 2009;11(1):R24. 10.1186/ar2617 19222863 PMC2688257

[32] Bienkowska J Allaire N Thai A: Lymphotoxin-LIGHT pathway regulates the interferon signature in rheumatoid arthritis. *PLoS One.* 2014;9(11):e112545. 10.1371/journal.pone.0112545 25405351 PMC4236099

[33] St Clair EW Baer AN Noaiseh G: The Clinical Efficacy and Safety of Baminercept, a lymphotoxin-Beta Receptor Fusion Protein, in Primary Sjögren’s Syndrome: Results from a Randomized, Double-Blind, Placebo-Controlled Phase II Trial.[Abstract 3203]. *Arthritis Rheumatol.* 2015;67(suppl 10). Reference Source 10.1002/art.40513 PMC6115299 29604186

[34] Hall JC Casciola-Rosen L Berger AE: Precise probes of type II interferon activity define the origin of interferon signatures in target tissues in rheumatic diseases. *Proc Natl Acad Sci U S A.* 2012;109(43):17609–14. 10.1073/pnas.1209724109 23045702 PMC3491524

[35] Hall JC Baer AN Shah AA: Molecular Subsetting of Interferon Pathways in Sjögren's Syndrome. *Arthritis Rheumatol.* 2015;67(9):2437–46. 10.1002/art.39204 25988820 PMC4551661

[36] Clancy RM Markham AJ Buyon JP: Endosomal Toll-like receptors in clinically overt and silent autoimmunity. *Immunol Rev.* 2016;269(1):76–84. 10.1111/imr.12383 26683146 PMC4685960

[37] Lövgren T Eloranta ML Båve U: Induction of interferon-alpha production in plasmacytoid dendritic cells by immune complexes containing nucleic acid released by necrotic or late apoptotic cells and lupus IgG. *Arthritis Rheum.* 2004;50(6):1861–72. 10.1002/art.20254 15188363

[38] Volkman HE Stetson DB: The enemy within: endogenous retroelements and autoimmune disease. *Nat Immunol.* 2014;15(5):415–22. 10.1038/ni.2872 24747712 PMC4131434

[39] Pender MP: Infection of autoreactive B lymphocytes with EBV, causing chronic autoimmune diseases. *Trends Immunol.* 2003;24(11):584–8. 10.1016/j.it.2003.09.005 14596882

[40] Iwakiri D: Multifunctional non-coding Epstein-Barr virus encoded RNAs (EBERs) contribute to viral pathogenesis. *Virus Res.* 2016;212:30–8. 10.1016/j.virusres.2015.08.007 26292159

[41] Hung T Pratt GA Sundararaman B: The Ro60 autoantigen binds endogenous retroelements and regulates inflammatory gene expression. *Science.* 2015;350(6259):455–9. 10.1126/science.aac7442 26382853 PMC4691329

[42] Croia C Astorri E Murray-Brown W: Implication of Epstein-Barr virus infection in disease-specific autoreactive B cell activation in ectopic lymphoid structures of Sjögren's syndrome. *Arthritis Rheumatol.* 2014;66(9):2545–57. 10.1002/art.38726 24891330

[43] Zeng M Hu Z Shi X: MAVS, cGAS, and endogenous retroviruses in T-independent B cell responses. *Science.* 2014;346(6216):1486–92. 10.1126/science.346.6216.1486 25525240 PMC4391621

[44] Fogel LA Yokoyama WM French AR: Natural killer cells in human autoimmune disorders. *Arthritis Res Ther.* 2013;15(4):216. 10.1186/ar4232 23856014 PMC3979027

[45] Rusakiewicz S Nocturne G Lazure T: NCR3/NKp30 contributes to pathogenesis in primary Sjogren's syndrome. *Sci Transl Med.* 2013;5(195):195ra96. 10.1126/scitranslmed.3005727 23884468 PMC4237161

[46] Dudakov JA Hanash AM van den Brink MR: Interleukin-22: immunobiology and pathology. *Annu Rev Immunol.* 2015;33:747–85. 10.1146/annurev-immunol-032414-112123 25706098 PMC4407497

[47] Ciccia F Guggino G Rizzo A: Potential involvement of IL-22 and IL-22-producing cells in the inflamed salivary glands of patients with Sjogren's syndrome. *Ann Rheum Dis.* 2012;71(2):295–301. 10.1136/ard.2011.154013 21979002

[48] Barone F Nayar S Campos J: IL-22 regulates lymphoid chemokine production and assembly of tertiary lymphoid organs. *Proc Natl Acad Sci U S A.* 2015;112(35):11024–9. 10.1073/pnas.1503315112 26286991 PMC4568258

[49] Devauchelle-Pensec V Mariette X Jousse-Joulin S: Treatment of primary Sjögren syndrome with rituximab: a randomized trial. *Ann Intern Med.* 2014;160(4):233–42. 10.7326/M13-1085 24727841

[50] Bowman S Everett C Bombardieri M: Preliminary Results of a Double-Blind Randomised Trial of Rituximab Anti-B-Cell Therapy in Patients with Primary Sjogrens Syndrome. [Abstract 11L]. *Arthritis Rheumatol.* 2015;67(suppl 10). Reference Source

[51] de Vita S Quartuccio L Seror R: THU0392 Efficacy and Safety of Belimumab Given for 12 Months in Primary sjögren's Syndrome: The Beliss Open-Label Phase II Study. *Ann Rheum Dis.* 2015;74(2):338–339. [Poster Presentation]. 10.1136/annrheumdis-2015-eular.3101

[52] Cornec D Costa S Devauchelle-Pensec V: Blood and salivary-gland BAFF-driven B-cell hyperactivity is associated to rituximab inefficacy in primary Sjögren's syndrome. *J Autoimmun.* 2016;67:102–10. 10.1016/j.jaut.2015.11.002 26688003

[53] Whang JA Chang BY: Bruton's tyrosine kinase inhibitors for the treatment of rheumatoid arthritis. *Drug Discov Today.* 2014;19(8):1200–4. 10.1016/j.drudis.2014.03.028 24721226

[54] Meiners PM Vissink A Kroese FG: Abatacept treatment reduces disease activity in early primary Sjögren's syndrome (open-label proof of concept ASAP study). *Ann Rheum Dis.* 2014;73(7):1393–6. 10.1136/annrheumdis-2013-204653 24473674

[55] Hu Y Metz DP Chung J: B7RP-1 blockade ameliorates autoimmunity through regulation of follicular helper T cells. *J Immunol.* 2009;182(3):1421–8. 10.4049/jimmunol.182.3.1421 19155489

[56] Saadoun D Rosenzwajg M Joly F: Regulatory T-cell responses to low-dose interleukin-2 in HCV-induced vasculitis. *N Engl J Med.* 2011;365(22):2067–77. 10.1056/NEJMoa1105143 22129253

[57] Long SA Rieck M Sanda S: Rapamycin/IL-2 combination therapy in patients with type 1 diabetes augments Tregs yet transiently impairs β-cell function. *Diabetes.* 2012;61(9):2340–8. 10.2337/db12-0049 22721971 PMC3425404

[58] Seror R Bootsma H Saraux A: Defining disease activity states and clinically meaningful improvement in primary Sjögren's syndrome with EULAR primary Sjögren's syndrome disease activity (ESSDAI) and patient-reported indexes (ESSPRI). *Ann Rheum Dis.* 2016;75(2):382–9. 10.1136/annrheumdis-2014-206008 25480887

[59] Devauchelle-Pensec V Gottenberg J Jousse-Joulin S: Which and How Many Patients Should Be Included in Randomised Controlled Trials to Demonstrate the Efficacy of Biologics in Primary Sjögren's Syndrome? *PLoS One.* 2015;10(9):e0133907. 10.1371/journal.pone.0133907 26368934 PMC4569343

[60] Oni C Mitchell S James K: Eligibility for clinical trials in primary Sjögren's syndrome: lessons from the UK Primary Sjögren's Syndrome Registry. *Rheumatology (Oxford).* 2016;55(3):544–52. 10.1093/rheumatology/kev373 26510429 PMC5854028

